# Improving Theory of Mind in Schizophrenia by Targeting Cognition and Metacognition with Computerized Cognitive Remediation: A Multiple Case Study

**DOI:** 10.1155/2017/7203871

**Published:** 2017-01-26

**Authors:** Élisabeth Thibaudeau, Caroline Cellard, Clare Reeder, Til Wykes, Hans Ivers, Michel Maziade, Marie-Audrey Lavoie, William Pothier, Amélie M. Achim

**Affiliations:** ^1^École de Psychologie, Université Laval, Québec, QC, Canada G1V 0A6; ^2^Centre de Recherche de l'Institut Universitaire en Santé Mentale de Québec, Québec, QC, Canada G1J 2G3; ^3^Institute of Psychiatry, Psychology and Neuroscience, King's College London, London SE5 8AF, UK; ^4^Département de Psychiatrie et Neurosciences, Faculté de Médecine, Université Laval, Québec, QC, Canada G1V 0A6

## Abstract

Schizophrenia is associated with deficits in theory of mind (ToM) (i.e., the ability to infer the mental states of others) and cognition. Associations have often been reported between cognition and ToM, and ToM mediates the relationship between impaired cognition and impaired functioning in schizophrenia. Given that cognitive deficits could act as a limiting factor for ToM, this study investigated whether a cognitive remediation therapy (CRT) that targets nonsocial cognition and metacognition could improve ToM in schizophrenia. Four men with schizophrenia received CRT. Assessments of ToM, cognition, and metacognition were conducted at baseline and posttreatment as well as three months and 1 year later. Two patients reached a significant improvement in ToM immediately after treatment whereas at three months after treatment all four cases reached a significant improvement, which was maintained through 1 year after treatment for all three cases that remained in the study. Improvements in ToM were accompanied by significant improvements in the most severely impaired cognitive functions at baseline or by improvements in metacognition. This study establishes that a CRT program that does not explicitly target social abilities can improve ToM.

## 1. Introduction

In schizophrenia, cognitive deficits are recognized as a core feature of the disorder [1–3], with 80% of patients exhibiting deficits in at least one cognitive domain [1, 4]. These deficits do not typically resolve following antipsychotic treatments [1, 2]. The impact of these deficits on interpersonal and occupational functioning is well-established [5, 6], leading to major efforts to further understand and treat these barriers to functional recovery. In this paper, we refer to cognition as including all nonsocial cognitive functions such as memory or executive functions. Along with cognitive deficits, patients with schizophrenia also present with social cognitive deficits [3]. Social cognition refers to the mental processes underlying social interactions, including the abilities involved in perceiving and interpreting social information in order to guide social interactions [7].

Among all cognitive and social cognitive functions, theory of mind (ToM) is most strongly associated with functioning in schizophrenia [5, 8–11] and is therefore an important treatment target. ToM can be defined as the ability to represent and infer the mental states of other people such as their intentions, emotions, or beliefs [7–9]. Even though ToM judgments are by definition social judgments, several nonsocial cognitive functions likely contribute to making correct inferences about other people's mental states. In real life, a poor memory could, for instance, make it harder to use relevant cues from previous encounters with the same person [12]. In line with this idea, several studies have reported significant associations between ToM and a range of cognitive abilities, including verbal memory, speed of processing, verbal fluency, cognitive flexibility, inhibition, and reasoning [13–21]. However, it remains unclear if one or a few cognitive functions are more strongly associated with ToM than the others. There is evidence that ToM acts as a mediator between cognition and different spheres of functioning such as social [9, 22, 23] or occupational functioning [9, 23, 24], with cognition influencing ToM, which in turn influences functioning. Therefore, ToM abilities may in fact be more proximal to functioning than cognitive abilities. Given that ToM is largely impaired in schizophrenia [3, 25–27], it is recognized as an important treatment target to promote better functioning in these patients.

Patients with schizophrenia often also present with metacognitive impairments, including difficulties in estimating the difficulty of a task (metacognitive knowledge) [28–30], monitoring their performance during a task, or regulating their cognition using efficient strategies (metacognitive regulation) [30]. Metacognitive skills are thought to contribute to ToM, for instance, by enabling the flexibility required to “shift back and forth from one's own perspective to the valid and differing perspectives of others” ([31], page 387). The improvement of metacognitive abilities could thus be useful for cognitive and social cognitive functioning.

Given the association between cognition and ToM abilities [13–21] and between metacognition and ToM [31–33], we could expect that addressing cognition and metacognition with a cognitive remediation therapy (CRT) could help improve ToM abilities in patients with schizophrenia. CRT is defined as a “behavioral training-based intervention that aims to improve cognitive processes with the goal of durability and generalization” [34]. Multiple CRT programs targeting various cognitive domains and using different methodology have been tested, revealing positive effects on cognitive performance (reviewed in [34, 35]). Other CRT programs targeting social cognition have led to improved social cognition performance (reviewed in [36]). However, despite the recognized relationship between cognition and ToM, no study has yet investigated if a CRT targeting only cognition and metacognition can also improve ToM performance. Furthermore, few studies have yet considered the cognitive profile at baseline (i.e., having a deficit in cognition or ToM) as an inclusion criterion. This in an important question given that cognitive deficits could act as a limiting factor for ToM.

The aim of this study was to investigate the effect of a CRT program (Computerized Interactive Remediation of Cognition Training for Schizophrenia; CIRCuiTS) that targets cognitive functions and metacognitive skills on ToM abilities in patients with schizophrenia. CIRCuiTS relies on a drill and strategy approach and puts a strong focus on the development of metacognitive skills and cognitive functions using nonemotive material. In addition, we also explored the changes in cognitive and metacognitive functions as well as clinical symptoms and global functioning following the CRT. We hypothesized that developing cognitive and metacognitive abilities with CIRCuiTS would lead to improvements in ToM.

## 2. Materials and Methods

### 2.1. Context

This multiple case study was conducted in parallel with a previous study [37] that assessed the feasibility of CIRCuiTS with young adults with schizophrenia. Four patients with ToM deficits at baseline were included in the present study, including two patients (Cases A and B) who were included in our previously published feasibility study (resp., designated as Cases A and C in the previous report [37]). ToM performance was assessed as part of the same study protocol, but these results were not previously published. None of the participants presented with a current developmental disorder that could have affected the performance, such as autism or attention deficit hyperactivity disorder.

### 2.2. Participants

All four participants had (1) a confirmed DSM-IV diagnosis of schizophrenia; (2) a duration of illness that did not exceed 10 years; (3) a clinical status that permitted reliable cognitive assessment (i.e., the patient did not present with acute psychotic symptoms that may affect the neuropsychological assessment and their psychiatrist considered that the collaboration and the medication were adequate for the patient); (4) cognitive difficulties defined as a performance equal to or below the 16th percentile either on the Rey Complex Figure Test [38] or on the California Verbal Learning Test-II [39]; and (5) a ToM deficit defined as a performance equal or below the 16th percentile on the Combined Stories Task (COST) [8].

Exclusion criteria for the study were (1) brain and metabolic disorders known to cause neuropsychological impairments; (2) substance dependence in the last six months; and (3) intellectual quotient (IQ) below 70 based on the Wechsler Adult Intelligence Scale third edition [40].

This study was approved by the ethics committee of the Centre de Recherche de l'Institut Universitaire en Santé Mentale de Québec in Québec, Canada, and all participants provided informed written consent.

### 2.3. Procedure

Baseline assessments included a ToM task and a battery of cognitive and metacognitive tasks, administered by a research assistant, as well as measures of clinical symptoms and global functioning, which were completed by the treating psychiatrist. Following baseline assessment, CRT was provided with the program CIRCuiTS, for a total of 40 sessions of approximately one hour each, at least three days per week (mean duration = 17.4 weeks, or about 4 months). The same assessment battery used at baseline was again administered after the end of the treatment. Furthermore, two follow-up assessments were conducted; the first one was conducted three months after the end of the treatment (and hence about 7 months after baseline assessment) and the second one was conducted 1 year after the end of the treatment (hence about 1 year and 4 months).

### 2.4. Material

#### 2.4.1. ToM Assessment

ToM was assessed with the Combined Stories Task (COST) [8], which requires participants to read short stories out loud and to answer one or two questions that require taking into account the characters' mental states (i.e., their intentions, beliefs, or emotions), for a total of 26 second-order ToM questions. Answers are rated as 0, 1, or 2 points for a total of 52 points. Participants are encouraged to refer back to the written story to answer the questions if they need, which is done to minimize the impact of potential difficulties in being attentive to the text or in remembering the story. Previous studies documented the excellent validity [8] and test-retest reliability [41] of the COST.

#### 2.4.2. Neuropsychological Assessment

The Wechsler Adult Intelligence Scale third edition was used to provide a full assessment of IQ (WAIS-III; [40]). The cognitive battery included tests assessing different functions addressed by CIRCuiTS: the digit span subtest (total) from the WAIS-III [40] for verbal working memory and the spatial span (total) [42] for visual working memory. Episodic memory (verbal and visual) was assessed using the long-delay free recall of the California Verbal Learning Test-II (CVLT-II) [39] and of the Rey Complex Figure Test (RCFT) [38]. The Continuous Performance Test-II (CPT-II) [43] was used to assess selective attention (omission and commission) and sustained attention (HRTBC and HSEBC). Cognitive flexibility was assessed with the Wisconsin Card Sorting Test, 128 cards (WCST) (number of categories completed) [44], the Stroop from the Delis-Kaplan Executive Function System [45] condition 3 (total time) was used to assess inhibition, the Tower of London (TOL) (total problems solved correctly with the minimum move) [46] was used to assess planning and organization, and the Matrix reasoning subtest from the WAIS-III [40] was used to assess reasoning. In the present study, a performance below the 16th percentile was considered as a deficit.

#### 2.4.3. Metacognitive Assessment

Metacognitive knowledge (i.e., the knowledge about one's own cognition) was assessed with the Subjective Scale to Investigate Cognition in Schizophrenia (SSTICS) [47]. This 21 items' questionnaire measures the patients' understanding of his own cognitive functioning [47]. The SSTICS score needs to be interpreted in the broader context of cognitive and behavioral performance. While an increased score can be interpreted as an increased impairment in these cognitive functions in daily life; it can also be interpreted like a better metacognitive knowledge about one's own cognition in daily life. Metacognitive regulation (i.e., the ability to monitor and regulate one's own cognition) was assessed with the Behavior Rating Inventory of Executive Function-Executive Global Index (BRIEF-A) [48]. This index includes a self and an informant report (i.e., a first-degree family member in the current study), and both were included in this protocol. The BRIEF-A can be used as an indicator of the patient awareness of his self-regulation [48]. A performance below the 16th percentile was considered as a deficit.

#### 2.4.4. Clinical Assessment

Global functioning was rated by the treating psychiatrist using the Global Assessment of Functioning (GAF) [49], which provides a single rating (0–100) encompassing psychological, social, and occupational functioning. Symptoms were rated with the Positive and Negative Syndrome Scale (PANSS) [50], which allows a distinction between the following five symptom factors: positive, negative, cognitive/disorganization, depression/anxiety, and excitability/hostility [51].

#### 2.4.5. CRT Program

The CRT program used in this study was the French Canadian adaptation of CIRCuiTS [37, 52, 53], an individual computerized CRT program that aims to improve cognition (attention, memory, and executive functions) and metacognitive skills. CIRCuiTS aims to improve metacognitive skills by promoting the constant monitoring, regulation, and revision of performance during a task [28]. The development of metacognitive skills is based on the metacognitive model presented by Wykes and Reeder [30] in which metacognitive knowledge and regulation are useful in the transfer of cognitive skills in everyday life. Metacognitive knowledge (knowledge about how cognition works in general and one's own cognition) can be helpful, for example, to remembering a grocery list by knowing that classifying items of a list in different categories facilitates remembering the information. Similarly, metacognitive regulation (monitoring and regulation of one's own cognition) can, for example, be used to adapt strategies in scheduling, such as “I struggle to remember my appointment, so I will set an alarm on my cellphone.”

This program trains cognitive functions using a drill/practice and strategies method using 27 different tasks, each with at least 12 levels of difficulty. A more complete description of the CRT program is provided elsewhere [37, 52].

### 2.5. Statistical Analyses

To investigate the effect of CIRCuiTS on ToM abilities, Reliable Change Indices (RCIs) were calculated for ToM performance as assessed with the COST. RCIs can be used to assess if the score of an individual has statistically changed after an intervention. They correspond to the difference between two measures of the same individual, divided by the standard error of the difference of the test [54, 55]:

(1) $$\begin{array}{c} RCI=\frac{x_{2}-x_{1}}{\sqrt{2{\left(s_{1\sqrt{1}-r_{xx}}\right)}^{2}}}. \end{array}$$

This figure is the equation published by Zahra and Hedge in 2010 based on Jacobson and Truax (1991) where *x*_1_ signifies a participant pretest score and *x*_2_ signifies the posttest score of the same participant. This score is divided by the Sdiff, or standard error of the change score, that is the difference between the scores (*x*_2_ and *x*_1_), corrected for the reliability of the instrument. The Sdiff represents the range of distribution of change scores that could be expected if no intervention was done and is further explained in Jacobson & Truax (1991). Briefly, *s*_1_ refers to the standard deviation of a normative group at baseline and *r*_*xx*_ refers to the test-retest reliability of the measure. Consequently, normative data were gathered from a comparative sample of *N* > 50 patients diagnosed with schizophrenia, and standard deviations were computed for each variable in order to estimate the SE in the RCI formula.

Here, an RCI was calculated (1) between baseline and posttreatment, (2) between baseline and three months after treatment, and (3) between baseline and 1 year after treatment. RCIs are very conservative and can be interpreted like *Z*-change scores, hence being considered statistically significant at *p* < 0.05 if equal or superior to 1.64 (one-tailed hypothesis) [54]. RCIs can be complemented by further calculating the clinical significance of the change, that is, whether the performance of a patient is in the range of the healthy control population or of the schizophrenia population after the treatment [56]. This is done by calculating a cut-off considering the means and standard deviations from a schizophrenia population and a healthy control population [56]. With this equation, the clinical cut-off score for the COST was 42.2/52. If the patient's score surpasses the cut-off point after the treatment, the change is considered clinically significant [56].

In addition to ToM, RCIs were also calculated for all cognitive and metacognitive measures, as well as for symptoms ratings and functioning ratings. To facilitate the interpretation of the RCIs for cognitive measures for which a decrease in scores reflects an improvement (CPT omission, commission, HRTBC, HSEBC, Stroop, BRIEF, SSTICS, and PANSS), these RCIs were multiplied by −1 such that positive RCIs always reflected improvements. To assess the clinical significance of changes in symptoms, changes of at least 25% on the PANSS total or subscale scores compared to baseline were considered as clinically significant, as proposed by Leucht [57]. For the GAF, a score above 59 was considered as a remission state, as proposed by Bertelsen et al. [58].

## 3. Results: Case Presentation

### 3.1. Case A

Case A is a 26-year-old man who was diagnosed with schizophrenia 7 years ago. At the time of testing, he was taking clozapine and lamotrigine and presented with mild to moderate positive (range of PANSS scores = 1–4; mean = 2.8) and cognitive/disorganization symptoms (range of PANSS scores = 1–5; mean = 2.8). He presented moderate difficulties in global functioning (GAF = 42). His symptom ratings at each time point are presented in the Supplementary Material (see Table S1 in Supplementary Material available online at https://doi.org/10.1155/2017/7203871). He completed 12 years of education and was working part-time and going to school full-time. His IQ at baseline was 78.

At baseline, Case A showed a severe ToM deficit, with a performance below the 1st percentile. Case A also showed cognitive deficits for visual and verbal episodic memory and cognitive flexibility and also showed impairment for the informant-rated metacognitive regulation. The scores and percentiles at each time point are presented in the Supplementary Material (Table S2). The evaluator reported that Case A did not seem aware of his cognitive deficits at the baseline. Case A completed 40 CRT sessions.

#### 3.1.1. Changes in ToM Performance

**Figure 1:**
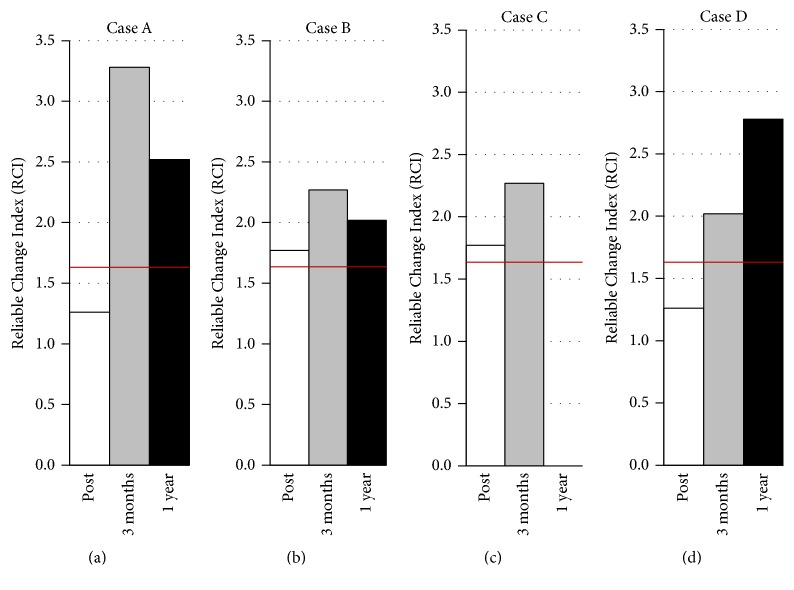
Reliable change indices (RCIs) for theory of mind (ToM) performance on the COST for the four cases. Post = RCI from baseline to posttreatment; 3 months = RCI from baseline to three months after treatment; 1 year = RCI from baseline to 1 year after treatment. The red lines indicate the statistical threshold of 1.64.

As presented in Figure 1(a), Case A showed an increase in ToM performance from baseline to posttreatment that did not reach statistical significance (RCI = 1.26). His performance further increased, and the improvement reached significance at three months after treatment (RCI = 3.28) and remained significant 1 year after treatment (RCI = 2.52). Regarding the clinical cut-off score, despite the observed improvements, Case A's ToM performance was still considered as clinically impaired.

#### 3.1.2. Concurrent Changes in Other Cognitive and Metacognitive Measures

**Figure 2:**
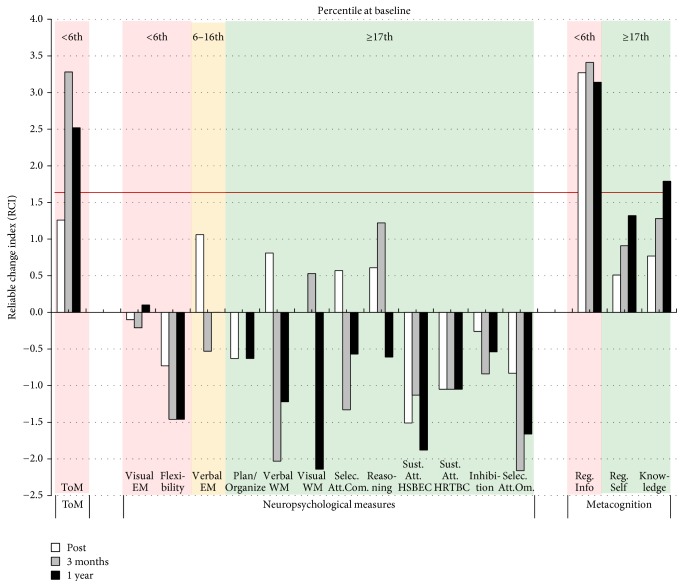
Changes in ToM and neuropsychological and metacognitive results for Case A for each of the follow-up assessments (posttreatment, 3 months and 1 year after treatment). The color of the background indicates the severity of the deficit at baseline. A red background indicates a severe impairment at baseline (≤6th percentile), a yellow background indicates an important impairment (7th to 16th percentile), and a green background indicates a normal performance (≥17th percentile). The horizontal red line indicates the statistical RCI threshold of 1.64. ToM = theory of mind; EM = episodic memory; WM = working memory; selec. att. = selective attention; com. = commission; sust. att. = sustained attention; HSBEC = Hit Standard Error Block Change; HRTBC = Hit Reaction Time Block Change; om. = omission; reg. info = Metacognitive Regulation Informant Report; reg. self = Metacognitive Regulation Self Report; knowledge = metacognitive knowledge.

As shown in Figure 2, no concurrent improvements in cognitive functions were observed following CRT. The evaluator noted that Case A seemed less motivated and that his attention was fluctuating throughout the posttreatment and the follow-up assessments. However, significant improvements were observed for metacognitive knowledge and informant-reported metacognitive regulation. Thus, Case A's significant improvements in ToM were mostly accompanied by improvements in his metacognitive abilities (see details in the Supplementary Material, Table S2).

#### 3.1.3. Concurrent Changes in Clinical Measures

Case A also showed a statistically and clinically significant improvement of his total clinical symptoms at posttreatment (RCI = 3.77), including his positive (RCI = 3.77), negative (RCI = 2.90), and depression/anxiety (RCI = 2.96) symptoms. The improvement in negative symptoms was the only one to remain clinically significant (though not statistically significant) at 3 months after treatment. There was a statistically significant improvement in functioning (RCI = 2.58) which could however be explained by the decrease of the severity of the clinical symptoms. This improvement in global functioning reached the remission criteria (59) at posttreatment, but these improvements did not last at the follow-up assessments (see details in the Supplementary Material, Table S1).

### 3.2. Case B

Case B is a 24-year-old man who was diagnosed with schizophrenia 6 years ago. At the time of testing, he was taking clozapine and presented with moderate to severe positive symptoms (range of PANSS score = 3–6; mean = 5.3) and moderate cognitive/disorganization symptoms (range of PANSS score = 1–6; mean = 4.0). Moderate difficulties were observed in his global functioning (GAF = 38). The symptoms ratings at each time point are presented in the Supplementary Material (Table S3). He completed 7 years of education and held a part-time job. His IQ at baseline was 73.

At baseline, Case B showed severe ToM impairments with a performance below the 1st percentile and his most important cognitive deficits were observed in episodic memory, cognitive flexibility, verbal working memory, and selective and sustained attention. The informant-rated metacognitive regulation was also impaired. The scores and percentiles at each time point are presented in the Supplementary Material (Table S4). Despite important delusions, Case B was collaborative and motivated during baseline assessment according to the evaluator. Case B completed 40 CRT sessions.

#### 3.2.1. Changes in ToM Performance

As presented in Figure 1(b), Case B showed a statistically significant improvement in ToM performance from baseline to posttreatment (RCI = 1.77). Case B again improved to reach his highest RCI three months after treatment (RCI = 2.27). His RCI also remained significant 1 year after treatment (RCI = 2.02). Regarding the clinical cut-off score, he was still considered as clinically impaired despite his improvements.

#### 3.2.2. Concurrent Changes in Other Cognitive and Metacognitive Measures

**Figure 3:**
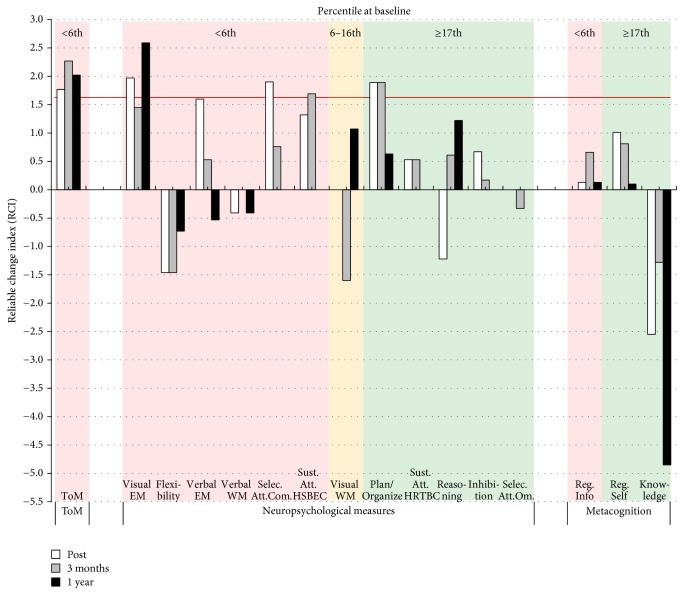
Changes in ToM and neuropsychological and metacognitive results for Case B for each of the follow-up assessments (posttreatment, 3 months and 1 year after treatment). The color of the background indicates the severity of the deficit at baseline. A red background indicates a severe impairment at baseline (≤6th percentile), a yellow background indicates an important impairment (7th to 16th percentile), and a green background indicates a normal performance (≥17th percentile). The horizontal red line indicates the statistical RCI threshold of 1.64. ToM = theory of mind; EM = episodic memory; WM = working memory; selec. att. = selective attention; com. = commission; sust. att. = sustained attention; HSBEC = Hit Standard Error Block Change; HRTBC = Hit Reaction Time Block Change; om. = omission; reg. info = Metacognitive Regulation Informant Report; reg. self = Metacognitive Regulation Self Report; knowledge = metacognitive knowledge.

As shown in Figure 3, Case B benefited from CRT, showing several cognitive improvements in areas impaired at baseline. Indeed, significant RCIs for the posttreatment and the two follow-up assessments are observed in visual episodic memory, selective and sustained attention, and planning/organization.

According to the evaluator, Case B was motivated at posttreatment assessment but his delusions interfered with the evaluation at three months and 1 year after treatment. No significant improvements were observed for the metacognitive measures (see details in Supplementary Material, Table S4).

#### 3.2.3. Concurrent Changes in Clinical Measures

Case B showed significant statistical improvements on the PANSS at posttreatment and for the two follow-ups for the total (RCI = 2.94; 2.66; 2.66), positive (RCI = 3.01; 1.88; 1.88), and cognitive/disorganization symptoms (RCI = 2.88; 2.88; 2.88). Changes were clinically significant for positive symptoms posttreatment. He showed statistically and clinically significant improvement of excitability/hostility symptoms three months and 1 year after treatment (RCI = 1.86; 1.86). No significant changes were observed for global functioning (see details in the Supplementary Material, Table S3).

### 3.3. Case C

Case C is a 28-year-old man who was diagnosed with schizophrenia 3 years ago. He was receiving clozapine and presented with moderate negative (range of PANSS score = 2–5; mean = 3.1) and moderate cognitive/disorganization symptoms (range of PANSS score = 1–4; mean = 2.2). Moderate difficulties were identified in his global functioning (GAF = 45). His symptom ratings at each time point are presented in the Supplementary Material (Table S5). He completed 11 years of education and was unemployed. His IQ at baseline was 75.

At baseline, Case C showed a large ToM deficit with a performance at the 8th percentile and his most important cognitive deficits were observed for visual episodic memory, selective attention, and executive functions (reasoning and cognitive flexibility). The scores and percentiles at each time point are presented in the Supplementary Material (Table S6). Despite showing good collaboration during the baseline assessment, Case C's attention seemed to fluctuate as reported by the evaluator. Case C completed 39 CRT sessions.

#### 3.3.1. Changes in ToM Performance

As presented in Figure 1(c), Case C showed a significant improvement in ToM performance from baseline to the end of the treatment (RCI = 1.77) and three months after treatment (RCI = 2.27).

Case C did not consent to participate to the last assessment 1 year after treatment. Case C had however already surpassed the clinical cut-off score at posttreatment, meaning that he was no longer considered as clinically impaired in ToM.

#### 3.3.2. Concurrent Changes in Other Cognitive and Metacognitive Measures

**Figure 4:**
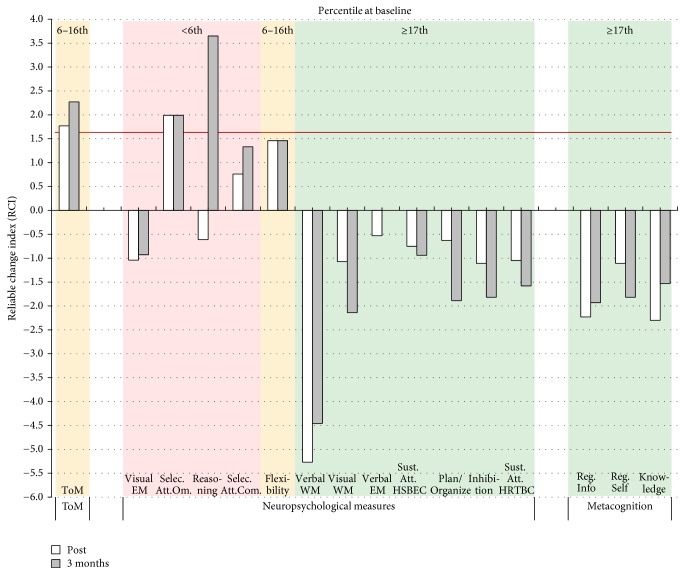
Changes in ToM and neuropsychological and metacognitive results for Case C for each of the follow-up assessments (posttreatment, 3 months and 1 year after treatment). The color of the background indicates the severity of the deficit at baseline. A red background indicates a severe impairment at baseline (≤6th percentile), a yellow background indicates an important impairment (7th to 16th percentile), and a green background indicates a normal performance (≥17th percentile). The horizontal red line indicates the statistical RCI threshold of 1.64. ToM = theory of mind; EM = episodic memory; selec. att. = selective attention; om. = omission; com. = commission; WM = working memory; sust. att. = sustained attention; HSBEC = Hit Standard Error Block Change; HRTBC = Hit Reaction Time Block Change; reg. info = Metacognitive Regulation Informant Report; reg. self = Metacognitive Regulation Self Report; knowledge = metacognitive knowledge.

As shown in Figure 4, Case C improved in cognitive functions that were impaired at baseline. Indeed, he showed a significant improvement in selective attention at posttreatment and three months after treatment, as well as a significant improvement in reasoning three months after treatment. No significant improvement was observed for the metacognitive measures (see details in the Supplementary Material, Table S6). The evaluator reported his impression that Case C exhibited a lack of motivation at posttreatment and during the three months' follow-up evaluation, which could have impacted his results.

#### 3.3.3. Concurrent Changes in Clinical Measures

The lack of motivation of Case C was not explained by a change in his clinical symptoms, which remained stable at posttreatment and during the three months' follow-up. The GAF score of Case C also remained stable (see details in the Supplementary Material, Table S5).

### 3.4. Case D

Case D is a 33-year-old man who was diagnosed with schizophrenia 2 years ago. At the time of testing, he was receiving clozapine and presented with moderate negative (range of PANSS score = 2–5; mean = 3.4) and cognitive/disorganization symptoms (range of PANSS score = 1–5; mean = 3.2) as well as mild depression/anxiety symptoms (range of PANSS score = 3-4; mean = 3.5). Moderate difficulties were present in his global functioning (GAF = 48). The ratings at each time point are presented in the Supplementary Material (Table S7). He completed 16 years of education and was unemployed. His IQ at baseline was 88.

At baseline, Case D showed a large ToM deficit with a performance corresponding to the 16th percentile. The most important cognitive deficits were observed for visual episodic memory, sustained attention, cognitive flexibility, and inhibition. Self-rated metacognitive regulation and metacognitive knowledge were also impaired. The scores and percentiles at each time point are presented in the Supplementary Material (Table S8). During baseline assessment, Case D showed good collaboration and motivation as reported by the evaluator. Case D completed 38 CRT sessions.

#### 3.4.1. Changes in ToM Performance

As presented in Figure 1(d), Case D showed a nonsignificant improvement in ToM performance from baseline to the end of treatment (RCI = 1.26) but then reached significant improvements three months after treatment (RCI = 2.02) and 1 year after treatment (RCI = 2.78). Regarding the clinical cut-off score, Case D surpassed the criterion and was no longer considered as clinically impaired at posttreatment and for the two follow-up assessments.

#### 3.4.2. Concurrent Changes in Other Cognitive and Metacognitive Measures

**Figure 5:**
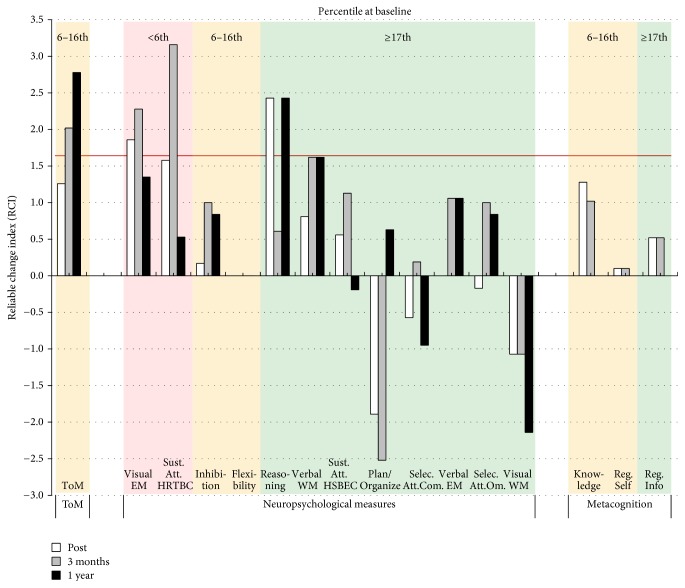
Changes in ToM and neuropsychological and metacognitive results for Case D for each of the follow-up assessments (posttreatment, 3 months and 1 year after treatment). The color of the background indicates the severity of the deficit at baseline. A red background indicates a severe impairment at baseline (≤6th percentile), a yellow background indicates an important impairment (7th to 16th percentile), and a green background indicates a normal performance (≥17th percentile). The horizontal red line indicates the statistical RCI threshold of 1.64. ToM = theory of mind; EM = episodic memory; sust. att. = sustained attention; HRTBC = Hit Reaction Time Block Change; WM = working memory; HSBEC = Hit Standard Error Block Change; selec. att. = selective attention; com. = commission; om. = omission; knowledge = metacognitive knowledge; reg. self = Metacognitive Regulation Self Report; reg. info = Metacognitive Regulation Informant Report.

As shown in Figure 5, Case D benefited from CRT with significant improvements in cognitive functioning, including visual episodic memory, sustained attention, and reasoning. No statistically significant improvements were observed for the metacognitive measures (see details in the Supplementary Material, Table S8). During the posttreatment and the follow-up assessments, the evaluator noted that Case D was doing his best and was taking a long time to complete the tasks properly.

#### 3.4.3. Concurrent Changes in Clinical Measures

A statistically and clinically significant change (RCI = 1.98) was observed for the depression/anxiety symptoms at posttreatment. A clinically significant (but not statistically significant) improvement was also observed for cognitive/disorganization symptoms. Data were not available three months and 1 year after treatment. The GAF score of Case D stayed stable at posttreatment (see details in the Supplementary Material, Table S7).

## 4. Discussion

The aim of this study was to investigate whether a CRT program (CIRCuiTS) that targets cognitive and metacognitive functions can lead to improvements in ToM abilities in patients with schizophrenia. Two out of four patients showed a statistically significant ToM improvement at the end of the treatment with CIRCuiTS, whereas at three months after treatment, the improvement was significant for all four patients. Data were also available at 1 year after treatment for three patients, and even after this prolonged period, the improvements remained significant for all three patients. Overall, the results of this multiple case study show that it is possible to improve ToM with a CRT program that targets nonsocial functions. Furthermore, the effect of this therapy on ToM lasted and even increased during the two follow-ups.

### 4.1. Cognitive Difficulties as a Limiting Factor for ToM Abilities

While the different processes that can be targeted to improve ToM still need to be better delineated, several studies have shown that ToM is an ability that relies on multiple social and nonsocial processes [9, 59–64]. As an example, to be able to identify the intention of another person, it might be useful to be attentive to social cues, to maintain, recall, and manipulate different information in a flexible way and to inhibit some responses. Associations between ToM and cognition are supported by several correlational studies that have shown significant relationship between ToM and various cognitive functions such as verbal memory [13, 15, 20] or executive functions [14, 19, 21]. Furthermore, Fanning et al. [13] reported that ToM deficits often present along with cognitive deficits in schizophrenia, such that 77% of patients that presented with ToM deficits also showed cognitive impairments in that study. The relationship between ToM and cognition is also supported by the literature in social neurosciences. Several studies have demonstrated that brain regions supporting ToM tasks are not specific to social stimuli, as they are also activated during nonsocial cognitive tasks [61, 64–66]. For instance, the temporoparietal junction is activated during attention reorientation tasks [64] and the medial prefrontal cortex during higher-level information integration tasks [61, 67]. However, despite multiple correlational studies, it remains unclear if certain cognitive functions are more crucial to ToM than others. While our results suggest that the improvement of severe cognitive deficits can help to improve ToM performance, this would need to be tested in a larger group before drawing any conclusions about the larger population of people with schizophrenia.

In the current study, the improvements in cognitive functions along with improvements in ToM are in agreement with the evidence in the literature that show associations between cognition and ToM. Three cases (B, C, and D) showed ToM improvements that were accompanied by significant improvements in the cognitive functions that were most severely impaired at baseline for each patient. The other case (A) who was less severely impaired in cognition showed significant improvement in metacognitive skills. Thus, our observations are consistent with the suggestion by Schaafsma et al. [59] that even if ToM tasks always involve social stimuli, several cognitive functions can contribute to attributing the correct mental states to another person. However, in order to develop targeted and efficient CRT treatment, a better understanding of the relationship between ToM, cognition, and metacognition is necessary.

**Figure 6:**
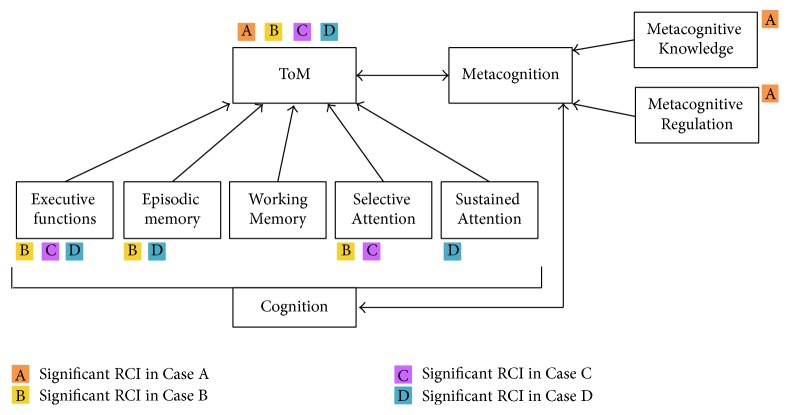
Possible targets leading to ToM improvement. The colors indicate a significant improvement following CRT. In this figure, executive functions include reasoning, cognitive flexibility, inhibition, and planning/organization. Episodic memory and working memory include visual and verbal modalities. Selective attention includes omissions and commissions. Sustained attention includes the change in reaction time.

While the processes at play remain to be identified, the current study supports our view that good cognitive functioning is a basis for ToM, as illustrated in Figure 6. Strengthening this basis thus seems like a relevant target to improve ToM and eventually functioning. For instance, to perform adequately on ToM task such as the COST or other story-based tasks, attentional and reasoning capacities are required, and deficits in these functions could impact the performance of the patient in ToM by reducing his ability to direct attention toward relevant information or by impairing the ability to make sense of a situation. The current study also suggests that metacognition can be useful to promote better ToM performance, which we also illustrate in Figure 6. According to the model of Wykes and Reeder [30], improvement of metacognitive knowledge and regulation enables the patient to be aware of his cognitive difficulties and to overcome these difficulties by the monitoring of his performance and the use of different strategies. The development of metacognition is often used in treatment, given that admitting a problem is recognized as an important first step to overcome it [32, 33]. On this topic, Passerieux et al. [33] suggested that, for two patients with the same level of cognitive deficit, the patient who has a better knowledge of his difficulties is in a better position to identify the challenges that it poses and to take action to minimize the impact of his deficits [33]. Thus, based on the literature and our results, we suggest that it is possible to improve ToM by targeting nonsocial cognitive deficits that act as a limiting factor for ToM. Also, we suggest that metacognitive abilities could promote not only cognitive functions, but also ToM performance. The current study therefore adds to the literature, providing evidence for the importance of cognition for ToM and the relevance of addressing these deficits in treatment for ToM abilities.

### 4.2. CRT to Improve ToM in Schizophrenia

Our results suggest that patients that present with multiple severe cognitive deficits at baseline may benefit more from a CRT that first targets general cognition, as these deficits could act as limiting factors for their ToM performance. In contrast, some patients with less cognitive deficits may be more likely to benefit from a CRT that directly targets their ToM abilities. CRTs designed specifically to improve ToM abilities either target one specific domain such as ToM [68, 69] or are broad-based and allow the training of different component of social cognition such as emotion recognition, attribution biases, and ToM [70, 71]. Furthermore, metacognition is a common target in CRT programs for social cognition to improve the knowledge that the patient has about his own difficulties [32, 33]. The development of metacognitive skills might be a key factor to improve ToM deficits as observed for Case A in the current study. However, except for Case A, who had severe impairment in metacognition at baseline, the other patients did not present with major deficits in metacognition. To improve their ToM abilities, patients are typically trained to direct their attention toward relevant information and interpret the meaning of different social situations presented in comic strips or videos [33]. Several CRT programs for ToM are based on strategy learning such as generating multiple hypotheses regarding a social situation, searching and selecting relevant cues, and developing intentional reasoning (i.e., which character does what and in what intention?) [33]. Thus, the development of strategies embedded in CIRCuiTS could be a key factor to improve ToM. CRT that target directly ToM has shown efficacy in patients with schizophrenia [72] but may not always be well-suited for all patients, or at least, not as a first treatment target.

People with schizophrenia present with heterogeneous cognitive profiles, with some patients showing impairments in several areas and others presenting with only mild deficits [1]. In order to enhance CRT efficacy, it is thus important to determine the variables underlying each individual's deficits and tailor the CRT to suit these specific deficits [73]. All patients start CRT with their own strengths and weaknesses, and it is increasingly recognized that better outcome is achieved when the treatment is personalized for a given patient based on his cognitive profile [37, 73, 74]. In fact, patients' cognitive profiles before the start of CRT has been reported as an important factor for treatment outcome in several studies [73, 75–77]. The specific improvements in cognition or metacognition observed in our study highlight the positive impact of the individualized approach of CIRCuiTS, which sets personal goals before and during the therapy to improve the most impaired cognitive functions for each patient. Thus, applying the exact same protocol of treatment to all patients might not be an optimal avenue to promote better cognitive and ToM performance. In light of the literature and the current results, it seems appropriate to acknowledge the cognitive profile of the patient before CRT and to adapt the treatment more specifically to his deficits, which may sometimes mean choosing a nonsocial CRT to improve ToM. In addition to the cognitive profile at baseline, several other variables could potentially influence the effect of CRT treatment, or how much treatment is required to observe an effect [73]. In a literature review, Medalia and colleagues [73] identified that factors that can promote a better response to CRT treatments include younger age (critical window given the higher neuroplasticity potential), shorter illness duration, increased number of CRT sessions, baseline cognitive reserve, motivation, perception of competence, and a good therapeutic alliance. In this study, patients who improved the most in ToM at posttest had a lower IQ, but no clear pattern was observed related to the age of the patients.

### 4.3. Detecting Cognitive Changes with Appropriate Measures

ToM tasks with good psychometric properties are essential to document changes in performance after CRT, but no standardized tasks are currently available. A major initiative [78] has recently investigated the psychometric properties of several promising social cognitive tasks and found that, for ToM, the Hinting task showed the strongest psychometric properties among tasks that they examined. However, some limitations such as ceiling effects or restricted ranges of performance have been observed for the Hinting task in previous studies [79, 80], which could limit the capacity of this task to detect changes after treatment.

The COST includes more items than the Hinting task and previous studies have revealed normal distributions even in healthy participants [8]. Previous studies have also shown good psychometric properties for the COST, including good convergent validity and internal consistency as well as excellent interrater reliability [8]. Furthermore, high test-retest reliability and an absence of a practice effect have been demonstrated in a previous study [41, 81, 82]. Thus, patients' improvements on the task in the current study are not likely due to repeated assessment of the task, though future studies will need to include a control group without treatment to control for such effects. Finally, the results of this study show that the COST is sensitive to changes, meaning that it is possible to detect a change in performance after a treatment. Therefore, the COST seems like an advantageous measure to document ToM improvements in future treatment studies.

In addition to taking the psychometric properties of the measures into account, it is important to carefully interpret the cognitive scores in the context of the CTR treatment that the patient received. For instance, visual examination of Figures 2, 3, 4, and 5 suggests that some patients in the present study showed* decreased* cognitive performance after CRT treatment, particularly for planning/organization and low-order cognitive functions such as working memory and attention. The task used to assess planning/organization involves solving a problem in a limited time. This is important because, during CIRCuiTS, patients are instructed to take their time and elaborate strategies when completing a task. Therefore, as proposed by Cellard et al. [83], a decrease in performance in this type of task should be interpreted with caution, as it could reflect a better use of strategies that impact positively everyday functioning. For low-order cognitive functions, decreases were mostly observed in patients for whom attention and working memory were not impaired at baseline whereas patients with severe impairment in these two cognitive functions improved after the therapy. This could reflect the personalized administration of CIRCuiTS in which impaired functions are more intensively trained. 

### 4.4. Limitations

The limitations of this study include that RCIs are very conservative and could prevent the detection of some clinical changes that could positively impact patient functioning, particularly at posttreatment, where several of the RCIs were close to the significance threshold. To address this limit, the clinically significant changes in ToM were also presented in the article, and the clinically significant changes for the cognitive and metacognitive measures were highlighted in the Supplementary Material. A second limitation is that it is not possible to perform statistical analyses of covariance in a multiple case study and we could therefore not directly test whether the improvements in ToM and in cognitive/metacognitive functions covary. Third, we cannot exclude the nonspecific effect of the treatment that could result from the patient having regular contact with the therapist, which could have helped improve his social skills. However, prior to CRT, these patients were routinely meeting with diverse clinicians. It would thus be surprising that meeting with the therapist for the CRT would more importantly impact ToM. Fourth, all patients were receiving clozapine, a medication for refractory clinical symptoms. It is however noteworthy that the psychiatrists who referred the four patients included in the current study are very proactive with the use of clozapine. While they respect the need for other prior treatments, they initiate clozapine as soon as a lack of response is detected. Patients had, however, generally responded well to clozapine and were stable at the time of the evaluation and CRT. This homogeneity of treatment could represent an advantage, though we cannot exclude that it might have interacted with the effect of the CRT. However, previous studies revealed no evidence of an effect of clozapine on cognition or ToM [84–87]. The inclusion of patients with more important metacognitive deficits at baseline would also be interesting to confirm the hypothesis that metacognition is a relevant target to improve ToM.

### 4.5. Conclusion

The results of this multiple case study provided initial evidence that a CRT targeting only nonsocial cognition and metacognition can significantly improve ToM abilities in patients with schizophrenia. These ToM improvements were mostly accompanied by improvements in the cognitive functions that were severely impaired at baseline, which for one case included metacognitive abilities. This is the first study, to the best of our knowledge, that has tested the effect of a nonsocial CRT on ToM. Though these results would certainly need to be replicated in a larger sample in order to determine if they can be more generally observed, they add to the literature by suggesting that cognitive deficits can act as a limiting factor for ToM at least in some patients. A better understanding of how good cognition and metacognition can promote ToM will be helpful in the future to ensure optimal treatment and to promote functional recovery in schizophrenia.

## Supplementary Material

Supplementary Material provides detailed results for ToM, neuropsychological, metacognitive and clinical measures, including raw scores, percentiles and RCIs for each case.
