# Supplementary material for: Improving Theory of Mind in Schizophrenia by Targeting Cognition and Metacognition with Computerized Cognitive Remediation: A Multiple Case Study
Source: Schizophr Res Treatment. 2017 Jan 26;2017:7203871. doi: 10.1155/2017/7203871 (PMC5299218; doi:10.1155/2017/7203871)
Supplement: Supplementary file 1 — Supplementary Material provides detailed results for ToM, neuropsychological, metacognitive and clinical measures, including raw scores, percentiles and RCIs for each case. [file 7203871.f1.zip › Supplementary material - Submission CRP 01-08-2016.docx]

**Supplementary material**

Table S1. Clinical information for Case A at baseline, post-treatment and the two follow-ups

|  |  | | Case A | | | | | | | | | |  | |  |
| --- | --- | --- | --- | --- | --- | --- | --- | --- | --- | --- | --- | --- | --- | --- | --- |
|  | | Baseline  raw  score | | Baseline  mean | Post-treatment  raw score | Post- treatment  mean | 3 months  raw  score | 3 months  mean | 1 year  raw  score | 1 year  mean | Baseline – post- treatment  RCI | Baseline – 3 months  RCI | | Baseline – 1 year  RCI | |
| GAF | | 42 | | - | **59** | - | 42 | - | 42 | - | **2.58*** | .00 | | .00 | |
| *PANSS*^a^ | |  | |  |  |  |  |  |  |  |  |  | |  | |
| Total | | 63 | |  | **36** | - | 57 | - | 60 | - | **3.77*** | .84 | | .42 | |
| Positive | | 17 | | 2.8/7 | **7** | 1.2/7 | 18 | 3/7 | 18 | 3/7 | **3.77*** | -.38 | | -.38 | |
| Negative | | 17 | | 2.4/7 | **8** | 1.1/7 | **12** | 1.7/7 | 14 | 2/7 | **2.90*** | 1.61 | | .97 | |
| Cognitive/Disorganization | | 14 | | 2.8/7 | 13 | 2.6/7 | 13 | 2.6/7 | 14 | 2.8/7 | .36 | .36 | | .00 | |
| Depression/Anxiety | | 10 | | 2.5/7 | **4** | 1/7 | 8 | 2/7 | 8 | 2/7 | **2.96*** | .99 | | .99 | |
| Excitability/Hostility | | 5 | | 1.3/7 | 4 | 1/7 | 6 | 1.5/7 | 6 | 1.5/7 | .47 | -.47 | | -.47 | |

* RCI ≥ 1.64

GAF = Global Assessment of Functioning; PANSS = Positive and Negative Syndrome Scale

^a^ PANSS RCIs are multiplied by -1. Therefore, a significant RCI indicates a decrease in the severity of the clinical symptoms.

Scores in bold for the PANSS indicate a clinical change after the treatment (a change of at least 25% compared to baseline). For the GAF, a score in bold indicates that the patient reached the remission criteria (59)

Table S2. ToM, cognition and metacognition raw scores, percentile ranks and RCIs for Case A at baseline, post-treatment and the two follow-ups

|  | Baseline  raw  score | Post-  treatment raw score | 3 months  raw  score | 1 year  raw  score | Baseline  percentile | Post-  treatment percentile | 3 months  percentile | 1 year  percentile | Baseline – post-treatment RCI | Baseline – 3 months RCI | Baseline – 1 year RCI |
| --- | --- | --- | --- | --- | --- | --- | --- | --- | --- | --- | --- |
| *A. Social cognition* |  |  |  |  |  |  |  |  |  |  |  |
| *ToM* - Combined Stories Task (/52) | 25 | 30 | 38 | 35 | <1 | .10 | 13 | 3 | 1.26 | **3.28*** | **2.52*** |
|  |  |  |  |  |  |  |  |  |  |  |  |
| *B. Cognition* |  |  |  |  |  |  |  |  |  |  |  |
| *Working memory* – Digit span | 16 | 18 | 11 | 13 | 25 | 50 | 5 | 9 | .81 | -2.03 | -1.22 |
| *Working memory* – Spatial span | 14 | 14 | 15 | 10 | 25 | 25 | 25 | 2 | .00 | .53 | -2.14 |
| *Episodic memory* – CVLT delayed recall | 7 | 9 | 6 | 7 | 7 | 16 | 2 | 2 | 1.06 | -.53 | .00 |
| *Episodic memory* – RCFT delayed recall | 6.5 | 6 | 5.5 | 7 | 1 | 1 | 1 | 1 | -.10 | -.21 | .10 |
| *Selective attention* – CPT omission | 0 | 5 | 13 | 10 | 79 | 24 | 1 | 1 | -.83 | -2.16 | -1.66 |
| *Selective attention* – CPT commission | 19 | 16 | 26 | 22 | 27 | 41 | 6 | 13 | .57 | -1.33 | -.57 |
| *Sustained attention* – Hit reaction time  Block change | .01 | .03 | .03 | .03 | 59 | 21 | 14 | 12 | -1.05 | -1.05 | -1.05 |
| *Sustained attention* – Hit standard error  Block change | .04 | .12 | .10 | .14 | 44 | 6 | 10 | 3 | -1.51 | -1.13 | -1.88 |
| *Reasoning* – Matrix^a^ | 9 | 10 | 11 | 8 | 37 | 50 | 63 | 25 | .61 | 1.22 | -.61 |
| *Cognitive flexibility* – WCST – Total  Categories | 3 | 2 | 1 | 1 | 2 | 1 | 1 | 1 | -.73 | -1.46 | -1.46 |
| *Inhibition* – Stroop 3 | 44.6 | 47.75 | 54.6 | 51 | 63 | 63 | 37 | 50 | -.26 | -.84 | -.54 |
| *Planning / Organization* – TOL – Total  Correct | 2 | 1 | 2 | 1 | 19 | 8 | 19 | 8 | -.63 | .00 | -.63 |
| *Metacognition regulation* – BRIEF self GEC^b^ | 56 | 51 | 47 | 43 | 27 | 47 | 63 | 75 | .51 | .91 | 1.32 |
| *Metacognition regulation* – BRIEF informant GEC^b^ | 89 | 64 | 63 | 65 | <.01 | 8 | 9 | 6 | **3.27*** | **3.41*** | **3.14*** |
| *Metacognition knowledge – SSTICS* | 21 | 18 | 16 | 14 | 62 | 73 | 79 | 83 | .77 | 1.28 | **1.79*** |

ToM = Theory of mind score of the Combined Stories Task; CVLT = California Verbal Learning Test –II; RCFT = Rey Complex Figure Test; CPT = Continuous Performance Test – II; WCST = Wisconsin Card Sorting Test -128 card; TOL = Tower Of London ; BRIEF – self GEC = Executive Global Index – self-report; BRIEF – informant GEC = Executive Global Index – informant report; SSTICS = Subjective Scale to Investigate Cognition in Schizophrenia

^a^ Scaled scores with a mean of 10 and a standard deviation of 3.

^b^T scores with a mean of 50 and a standard deviation of 10.

Percentiles in bold indicate a clinical change after the treatment for the cognitive and metacognitive measures (a percentile that surpasses the 16^th^ percentile compared to baseline)

* RCI ≥ 1.64

Table S3. Clinical information for Case B at baseline, post-treatment and the two follow-ups

|  | Case B | | | | | | | | | | |  | |  |
| --- | --- | --- | --- | --- | --- | --- | --- | --- | --- | --- | --- | --- | --- | --- |
|  | | Baseline  raw  score | Baseline  mean | Post- treatment  raw score | Post- treatment  mean | 3 months  raw score | 3 months  mean | 1 year  raw score | 1 year  mean | Baseline – post- treatment  RCI | Baseline – 3 months  RCI | | Baseline – 1 year  RCI | |
| GAF | | 38 | - | 40 | - | 43 | - | 43 | - | .30 | .76 | | .76 | |
| *PANSS^a^* | |  |  |  |  |  |  |  |  |  |  | |  | |
| Total | | 87 | 3.29/7 | 66 | - | 68 | - | 68 | - | **2.94*** | **2.66*** | | **2.66*** | |
| Positive | | 32 | 5.3/7 | **24** | 4/7 | 27 | 4.5/7 | 27 | 4.5/7 | **3.01*** | **1.88*** | | **1.88*** | |
| Negative | | 19 | 2.7/7 | 18 | 2.6/7 | 18 | 2.6/7 | 18 | 2.6/7 | .32 | .32 | | .32 | |
| Cognitive/Disorganization | | 20 | 4/7 | **12** | 2.4/7 | **12** | 2.4/7 | **12** | 2.4/7 | **2.88*** | **2.88*** | | **2.88*** | |
| Depression/Anxiety | | 8 | 2/7 | 7 | 1.8/7 | 7 | 1.8/7 | 7 | 1.8/7 | .49 | .49 | | .49 | |
| Excitability/Hostility | | 8 | 2/7 | **5** | 1.3/7 | **4** | 1/7 | **4** | 1/7 | 1.40 | **1.86*** | | **1.86*** | |

* RCI ≥ 1.64

GAF = Global Assessment of Functioning; PANSS = Positive and Negative Syndrome Scale

^a^ PANSS RCIs are multiplied by -1. Therefore, a significant RCI indicates a decrease in the severity of the clinical symptoms.

Scores in bold for the PANSS indicate a clinical change after the treatment (a change of at least 25% compared to baseline). For the GAF, a score in bold indicates that the patient reached the remission criteria (59)

Table S4. ToM, cognition and metacognition raw scores, percentile ranks and RCIs for Case B at baseline, post-treatment and the two follow-ups

|  | Baseline  raw  score | Post-  treatment raw score | 3 months  raw  score | 1 year  raw  score | Baseline  Percentile | Post –  treatment percentile | 3 months  percentile | 1 year  Percentile | Baseline – post- treatment RCI | Baseline – 3 months RCI | Baseline –  1 year  RCI |
| --- | --- | --- | --- | --- | --- | --- | --- | --- | --- | --- | --- |
| *A. Social cognition* |  |  |  |  |  |  |  |  |  |  |  |
| *ToM* - Combined Stories Task (/52) | 23 | 30 | 32 | 31 | <.10 | .10 | 1 | .20 | **1.77*** | **2.27*** | **2.02*** |
|  |  |  |  |  |  |  |  |  |  |  |  |
| *B. Cognition* |  |  |  |  |  |  |  |  |  |  |  |
| *Working memory* – Digit span | 12 | 11 | 12 | 11 | 5 | 5 | 9 | 5 | -.41 | .00 | -.41 |
| *Working memory* – Spatial span | 13 | 13 | 10 | 15 | 16 | 16 | 2 | **25** | .00 | -1.60 | 1.07 |
| *Episodic memory* – CVLT delayed recall | 6 | 9 | 7 | 5 | 2 | 16 | 7 | 1 | 1.60 | .53 | -.53 |
| *Episodic memory* – RCFT delayed recall | 7 | 16.5 | 14 | 19.5 | 1 | 4 | 1 | 14 | **1.97*** | 1.45 | **2.59*** |
| *Selective attention* – CPT omission | 1 | 1 | 3 | NA | 70 | 70 | 46 | NA | .00 | -.33 | NA |
| *Selective attention* – CPT commission | 27 | 17 | 23 | NA | 5 | **36** | 13 | NA | **1.90*** | .76 | NA |
| *Sustained attention* – Hit reaction time  Block change | .02 | .01 | .01 | NA | 23 | 32 | 43 | NA | .53 | .53 | NA |
| *Sustained attention* – Hit standard error  Block change | .13 | .06 | .04 | NA | 5 | **19** | **29** | NA | 1.32 | **1.69*** | NA |
| *Reasoning* – Matrix^a^ | 9 | 7 | 10 | 11 | 37 | 16 | 50 | 63 | -1.22 | .61 | 1.22 |
| *Cognitive flexibility* – WCST – Total  Categories | 2 | .00 | .00 | 1 | 1 | 1 | 1 | 1 | -1.46 | -1.46 | -.73 |
| *Inhibition* – Stroop 3 | 48 | 40 | 46 | 48 | 63 | 84 | 63 | 63 | .67 | .17 | .00 |
| *Planning / Organization* – TOL – Total  Correct | 2 | 5 | 5 | 3 | 19 | 60 | 60 | 33 | **1.89*** | **1.89*** | .63 |
| *Metacognition regulation* – BRIEF self GEC^b^ | 54 | 44 | 46 | 53 | 33 | 72 | 66 | 37 | 1.01 | .81 | .10 |
| *Metacognition regulation* – BRIEF informant GEC^b^ | 65 | 64 | 60 | 64 | 6 | 8 | 16 | 8 | .13 | .66 | .13 |
| *Metacognition knowledge – SSTICS* | 15 | 25 | 20 | 34 | 81 | 52 | 66 | 27 | -2.55 | -1.28 | -4.85 |

ToM = Theory of mind score of the Combined Stories Task; CVLT = California Verbal Learning Test –II; RCFT = Rey Complex Figure Test; CPT = Continuous Performance Test – II; WCST = Wisconsin Card Sorting Test -128 card; TOL = Tower Of London ; BRIEF – self GEC = Executive Global Index – self-report; BRIEF – informant GEC = Executive Global Index – informant report; SSTICS = Subjective Scale to Investigate Cognition in Schizophrenia

^a^ Scaled scores with a mean of 10 and a standard deviation of 3.

^b^T scores with a mean of 50 and a standard deviation of 10.

Percentiles in bold indicate a clinical change after the treatment for the cognitive and metacognitive measures (a percentile that surpasses the 16^th^ percentile compared to baseline)

* RCI ≥ 1.64

Table S5. Clinical information for Case C at baseline, post-treatment and the two follow-ups

|  | Case C | | | | | | | | | | |  | |  |
| --- | --- | --- | --- | --- | --- | --- | --- | --- | --- | --- | --- | --- | --- | --- |
|  | | Baseline  raw score | Baseline  mean | Post- treatment  raw score | Post- treatment  mean | 3 months  raw score | 3 months  mean | 1 year  raw  score | 1 year  mean | Baseline – post- treatment RCI | Baseline – 3 months RCI | | Baseline – 1 year  RCI | |
| GAF | | 45 | - | 45 | - | 45 | - | NA | - | .00 | .00 | | NA | |
| *PANSS*^a^ | |  |  |  |  |  |  |  |  |  |  | |  | |
| Total | | 52 | 1.9/7 | 49 | - | 49 | - | NA | - | .42 | .42 | | NA | |
| Positive | | 8 | 1.3/7 | 8 | 1.3/7 | 8 | 1.3/7 | NA | NA | .00 | .00 | | NA | |
| Negative | | 22 | 3.1/7 | 20 | 2.9/7 | 20 | 2.9/7 | NA | NA | .65 | .65 | | NA | |
| Cognitive/Disorganization | | 11 | 2.2/7 | 10 | 2/7 | 10 | 2/7 | NA | NA | .36 | .36 | | NA | |
| Depression/Anxiety | | 7 | 1.8/7 | 7 | 1.8/7 | 7 | 1.8/7 | NA | NA | .00 | .00 | | NA | |
| Excitability/Hostility | | 4 | 1/7 | 4 | 1/7 | 4 | 1/7 | NA | NA | .00 | .00 | | NA | |

* RCI ≥ 1.64

GAF = Global Assessment of Functioning; PANSS = Positive and Negative Syndrome Scale

^a^ PANSS RCIs are multiplied by -1. Therefore, a significant RCI indicates a decrease in the severity of the clinical symptoms.

Scores in bold for the PANSS indicate a clinical change after the treatment (a change of at least 25% compared to baseline). For the GAF, a score in bold indicates that the patient reached the remission criteria (59)

Table S6. ToM, cognition and metacognition raw scores, percentile ranks and RCIs for Case C at baseline, post-treatment and the two follow-ups

|  | Baseline  raw  score | Post-  treatment raw score | 3 months  raw  score | 1 year  raw  score | Baseline  percentile | Post-  treatment percentile | 3 months  percentile | 1 year  percentile | Baseline – post-treatment RCI | Baseline – 3 months RCI | Baseline –  1 year  RCI |
| --- | --- | --- | --- | --- | --- | --- | --- | --- | --- | --- | --- |
| *A. Social cognition* |  |  |  |  |  |  |  |  |  |  |  |
| *ToM* - Combined Stories Task (/52) | 37 | 44 | 46 | NA | 8 | 51 | 51 | NA | **1.77*** | **2.27*** | NA |
|  |  |  |  |  |  |  |  |  |  |  |  |
| *B. Cognition* |  |  |  |  |  |  |  |  |  |  |  |
| *Working memory* – Digit span | 28 | 15 | 17 | NA | 25 | 25 | 37 | NA | -5.27 | -4.46 | NA |
| *Working memory* – Spatial span | 15 | 13 | 11 | NA | 25 | 16 | 5 | NA | -1.07 | -2.14 | NA |
| *Episodic memory* – CVLT delayed recall | 11 | 10 | 11 | NA | 32 | 7 | 50 | NA | -.53 | .00 | NA |
| *Episodic memory* – RCFT delayed recall | 8 | 3 | 3.5 | NA | 1 | 1 | 1 | NA | -1.04 | -.93 | NA |
| *Selective attention* – CPT omission | 17 | 5 | 5 | NA | 1 | **24** | **24** | NA | **1.99*** | **1.99*** | NA |
| *Selective attention* – CPT commission | 27 | 23 | 20 | NA | 5 | 13 | **23** | NA | .76 | 1.33 | NA |
| *Sustained attention* – Hit reaction time  Block change | -.02 | .00 | .01 | NA | 77 | 51 | 38 | NA | -1.05 | -1.58 | NA |
| *Sustained attention* – Hit standard error  Block change | .02 | .06 | .07 | NA | 44 | 22 | 19 | NA | -.75 | -.94 | NA |
| *Reasoning* – Matrix^a^ | 4 | 3 | 10 | NA | 2 | 1 | **50** | NA | -.61 | **3.65*** | NA |
| *Cognitive flexibility* – WCST – Total  Categories | 4 | 6 | 6 | NA | 11 | **16** | **16** | NA | 1.46 | 1.46 | NA |
| *Inhibition* – Stroop 3 | 50 | 47 | 53 | NA | 50 | 63 | 37 | NA | .25 | -.25 | NA |
| *Planning / Organization* – TOL – Total  Correct | 4 | 3 | 1 | NA | 47 | 33 | 8 | NA | -.63 | -1.89 | NA |
| *Metacognition regulation* – BRIEF self GEC^b^ | 50 | 61 | 68 | NA | 50 | 14 | 3 | NA | -1.11 | -1.82 | NA |
| *Metacognition regulation* – BRIEF informant GEC^b^ | 51 | 68 | 66 | NA | 47 | 3 | 5 | NA | -2.23 | -1.96 | NA |
| *Metacognition knowledge – SSTICS* | 2 | 11 | 8 | NA | 97 | 86 | 90 | NA | -2.30 | -1.53 | NA |

ToM = Theory of mind score of the Combined Stories Task; CVLT = California Verbal Learning Test –II; RCFT = Rey Complex Figure Test; CPT = Continuous Performance Test – II; WCST = Wisconsin Card Sorting Test -128 card; TOL = Tower Of London ; BRIEF – self GEC = Executive Global Index – self-report; BRIEF – informant GEC = Executive Global Index – informant report; SSTICS = Subjective Scale to Investigate Cognition in Schizophrenia

^a^ Scaled scores with a mean of 10 and a standard deviation of 3.

^b^T scores with a mean of 50 and a standard deviation of 10.

Percentiles in bold indicate a clinical change after the treatment for the cognitive and metacognitive measures (a percentile that surpasses the 16^th^ percentile compared to baseline)

* RCI ≥ 1.64

Table S7. Clinical information for Case D at baseline, post-treatment and the two follow-ups

|  | Case D | | | | | | | | | | |  | |  |
| --- | --- | --- | --- | --- | --- | --- | --- | --- | --- | --- | --- | --- | --- | --- |
|  | | Baseline  raw  score | Baseline  mean | Post- treatment  raw score | Post- treatment  mean | 3 months  raw score | 3 months  mean | 1 year  raw score | 1 year  mean | Baseline – post- treatment RCI | Baseline – 3 months RCI | | Baseline – 1 year  RCI | |
| GAF | | 48 | - | 48 | - | NA | NA | NA | NA | .00 | NA | | NA | |
| *PANSS*^a^ | |  |  |  |  |  |  |  |  |  |  | |  | |
| Total | | 71 | 2.7/7 | 62 | - | NA | NA | NA | NA | 1.26 | NA | | NA | |
| Positive | | 13 | 2.2/7 | 14 | 2.3/7 | NA | NA | NA | NA | -.38 | NA | | NA | |
| Negative | | 24 | 3.4/7 | 22 | 3.1/7 | NA | NA | NA | NA | .65 | NA | | NA | |
| Cognitive/Disorganization | | 16 | 3.2/7 | **12** | 2.4/7 | NA | NA | NA | NA | 1.44 | NA | | NA | |
| Depression/Anxiety | | 14 | 3.5/7 | **10** | 2.5/7 | NA | NA | NA | NA | **1.98*** | NA | | NA | |
| Excitability/Hostility | | 4 | 1/7 | 4 | 1/7 | NA | NA | NA | NA | .00 | NA | | NA | |

* RCI ≥ 1.64

GAF = Global Assessment of Functioning; PANSS = Positive and Negative Syndrome Scale

^a^ PANSS RCIs are multiplied by -1. Therefore, a significant RCI indicates a decrease in the severity of the clinical symptoms.

Scores in bold for the PANSS indicate a clinical change after the treatment (a change of at least 25% compared to baseline). For the GAF, a score in bold indicates that the patient reached the remission criteria (59)

Table S8. ToM, cognition and metacognition raw scores, percentile ranks and RCIs for Case D at baseline, post-treatment and the two follow-ups

|  | Baseline  raw  score | Post-  treatment raw score | 3 months  raw  score | 1 year  raw  Score | Baseline  percentile | Post-  treatment percentile | 3 months  percentile | 1 year  percentile | Baseline – post-treatment RCI | Baseline – 3 months RCI | Baseline – 1 year  RCI |
| --- | --- | --- | --- | --- | --- | --- | --- | --- | --- | --- | --- |
| *A. Social cognition* | 39 | 44 | 47 | 50 | 16 | 51 | 75 | 91 | 1.26 | **2.02*** | **2.78*** |
| *ToM* - Combined Stories Task (/52) |  |  |  |  |  |  |  |  |  |  |  |
|  |  |  |  |  |  |  |  |  |  |  |  |
| *B. Cognition* |  |  |  |  |  |  |  |  |  |  |  |
| *Working memory* – Digit span | 16 | 18 | 20 | 20 | 37 | 50 | 75 | 75 | .81 | 1.62 | 1.62 |
| *Working memory* – Spatial span | 20 | 18 | 18 | 16 | 84 | 63 | 63 | 50 | -1.07 | -1.07 | -2.14 |
| *Episodic memory* – CVLT delayed recall | 14 | 14 | 16 | 16 | 70 | 70 | 94 | 93 | .00 | 1.06 | 1.06 |
| *Episodic memory* – RCFT delayed recall | 11 | 20 | 22 | 17.5 | 1 | **24** | **38** | 10 | **1.86*** | **2.28*** | 1.35 |
| *Selective attention* – CPT omission | 0 | 1 | 1 | .00 | 79 | 70 | 70 | 79 | -.17 | -.17 | .00 |
| *Selective attention* – CPT commission | 11 | 14 | 10 | 16 | 63 | 48 | 71 | 35 | -.57 | .19 | -.95 |
| *Sustained attention* – Hit reaction time  Block change | .05 | .02 | -.01 | .04 | 3 | **25** | **62** | 9 | 1.58 | **3.16*** | .53 |
| *Sustained attention* – Hit standard error  Block change | .03 | .00 | -.03 | .04 | 38 | 53 | 62 | 33 | .56 | 1.13 | -.19 |
| *Reasoning* – Matrix^a^ | 9 | 13 | 10 | 13 | 37 | 84 | 50 | 84 | **2.43*** | .61 | **2.43*** |
| *Cognitive flexibility* – WCST – Total  Categories | 6 | 6 | 6 | 6 | 16 | 16 | 16 | 16 | .00 | .00 | .00 |
| *Inhibition* – Stroop 3 | 66 | 64 | 54 | 56 | 16 | 16 | **50** | **37** | .17 | 1.00 | .84 |
| *Planning / Organization* – TOL – Total  Correct | 4 | 1 | .00 | 5 | 40 | 9 | 1 | 51 | -1.89 | -2.52 | .63 |
| *Metacognition regulation* – BRIEF self GEC^b^ | 60 | 59 | 59 | NA | 16 | **18** | **18** | NA | .10 | .10 | NA |
| *Metacognition regulation* – BRIEF informant GEC^b^ | 56 | 52 | 52 | NA | 27 | 45 | 45 | NA | .52 | .52 | NA |
| *Metacognition knowledge – SSTICS* | 41 | 36 | 37 | NA | 14 | **19** | **18** | NA | 1.28 | 1.02 | NA |

ToM = Theory of mind score of the Combined Stories Task; CVLT = California Verbal Learning Test –II; RCFT = Rey Complex Figure Test; CPT = Continuous Performance Test – II; WCST = Wisconsin Card Sorting Test -128 card; TOL = Tower Of London ; BRIEF – self GEC = Executive Global Index – self-report; BRIEF – informant GEC = Executive Global Index – informant report; SSTICS = Subjective Scale to Investigate Cognition in Schizophrenia

^a^ Scaled scores with a mean of 10 and a standard deviation of 3.

^b^T scores with a mean of 50 and a standard deviation of 10.

Percentiles in bold indicate a clinical change after the treatment for the cognitive and metacognitive measures (a percentile that surpasses the 16^th^ percentile compared to baseline)

* RCI ≥ 1.64
