# Supplementary material for: Improving Theory of Mind in Schizophrenia by Targeting Cognition and Metacognition with Computerized Cognitive Remediation: A Multiple Case Study
Source: Schizophr Res Treatment. 2017 Jan 26;2017:7203871. doi: 10.1155/2017/7203871 (PMC5299218; doi:10.1155/2017/7203871)
Supplement: Supplementary file 1 — Supplementary Material provides detailed results for ToM, neuropsychological, metacognitive and clinical measures, including raw scores, percentiles and RCIs for each case. [file 7203871.f1.zip › Supplementary material - Submission CRP 01-08-2016.pdf]

Supplementary material

Table S1. Clinical information for Case A at baseline, post-treatment and the two follow-ups

	Case A										
	Baseline raw score	Baseline mean	Post-treatment raw score	Post-treatment mean	3 months raw score	3 months mean	1 year raw score	1 year mean	Baseline – post-treatment RCI	Baseline – 3 months RCI	Baseline – 1 year RCI
GAF	42	-	**59**	-	42	-	42	-	**2.58***	.00	.00
PANSS[a]											
Total	63		**36**	-	57	-	60	-	**3.77***	.84	.42
Positive	17	2.8/7	**7**	1.2/7	18	3/7	18	3/7	**3.77***	-.38	-.38
Negative	17	2.4/7	**8**	1.1/7	**12**	1.7/7	14	2/7	**2.90***	1.61	.97
Cognitive/Disorganization	14	2.8/7	13	2.6/7	13	2.6/7	14	2.8/7	.36	.36	.00
Depression/Anxiety	10	2.5/7	**4**	1/7	8	2/7	8	2/7	**2.96***	.99	.99
Excitability/Hostility	5	1.3/7	4	1/7	6	1.5/7	6	1.5/7	.47	-.47	-.47

* RCI ≥ 1.64

GAF = Global Assessment of Functioning; PANSS = Positive and Negative Syndrome Scale

[a] PANSS RCIs are multiplied by -1. Therefore, a significant RCI indicates a decrease in the severity of the clinical symptoms.

Scores in bold for the PANSS indicate a clinical change after the treatment (a change of at least 25% compared to baseline). For the GAF, a score in bold indicates that the patient reached the remission criteria (59)

Table S2. ToM, cognition and metacognition raw scores, percentile ranks and RCIs for Case A at baseline, post-treatment and the two follow-ups

	Baseline raw score	Post-treatment raw score	3 months raw score	1 year raw score	Baseline percentile	Post-treatment percentile	3 months percentile	1 year percentile	Baseline – post-treatment RCI	Baseline – 3 months RCI	Baseline – 1 year RCI
A. Social cognition											
ToM - Combined Stories Task (/52)	25	30	38	35	<1	.10	13	3	1.26	**3.28***	**2.52***
B. Cognition											
Working memory – Digit span	16	18	11	13	25	50	5	9	.81	-2.03	-1.22
Working memory – Spatial span	14	14	15	10	25	25	25	2	.00	.53	-2.14
Episodic memory – CVLT delayed recall	7	9	6	7	7	16	2	2	1.06	-.53	.00
Episodic memory – RCFT delayed recall	6.5	6	5.5	7	1	1	1	1	-.10	-.21	.10
Selective attention – CPT omission	0	5	13	10	79	24	1	1	-.83	-2.16	-1.66
Selective attention – CPT commission	19	16	26	22	27	41	6	13	.57	-1.33	-.57
Sustained attention – Hit reaction time Block change	.01	.03	.03	.03	59	21	14	12	-1.05	-1.05	-1.05
Sustained attention – Hit standard error Block change	.04	.12	.10	.14	44	6	10	3	-1.51	-1.13	-1.88
Reasoning – Matrix[a]	9	10	11	8	37	50	63	25	.61	1.22	-.61
Cognitive flexibility – WCST – Total Categories	3	2	1	1	2	1	1	1	-.73	-1.46	-1.46
Inhibition – Stroop 3	44.6	47.75	54.6	51	63	63	37	50	-.26	-.84	-.54
Planning / Organization – TOL – Total Correct	2	1	2	1	19	8	19	8	-.63	.00	-.63
Metacognition regulation – BRIEF self GEC[b]	56	51	47	43	27	47	63	75	.51	.91	1.32
Metacognition regulation – BRIEF informant GEC[b]	89	64	63	65	<.01	8	9	6	**3.27***	**3.41***	**3.14***
Metacognition knowledge – SSTICS	21	18	16	14	62	73	79	83	.77	1.28	**1.79***

ToM = Theory of mind score of the Combined Stories Task; CVLT = California Verbal Learning Test –II; RCFT = Rey Complex Figure Test; CPT = Continuous Performance Test – II; WCST = Wisconsin Card Sorting Test -128 card; TOL = Tower Of London ; BRIEF – self GEC = Executive Global Index – self-report; BRIEF – informant GEC = Executive Global Index – informant report; SSTICS = Subjective Scale to Investigate Cognition in Schizophrenia

[a] Scaled scores with a mean of 10 and a standard deviation of 3.

[b]T scores with a mean of 50 and a standard deviation of 10.

Percentiles in bold indicate a clinical change after the treatment for the cognitive and metacognitive measures (a percentile that surpasses the 16th percentile compared to baseline)

* RCI ≥ 1.64

Table S3. Clinical information for Case B at baseline, post-treatment and the two follow-ups

	Case B										
	Baseline raw score	Baseline mean	Post-treatment raw score	Post-treatment mean	3 months raw score	3 months mean	1 year raw score	1 year mean	Baseline – post-treatment RCI	Baseline – 3 months RCI	Baseline – 1 year RCI
GAF	38	-	40	-	43	-	43	-	.30	.76	.76
PANSS[a]											
Total	87	3.29/7	66	-	68	-	68	-	**2.94***	**2.66***	**2.66***
Positive	32	5.3/7	**24**	4/7	27	4.5/7	27	4.5/7	**3.01***	**1.88***	**1.88***
Negative	19	2.7/7	18	2.6/7	18	2.6/7	18	2.6/7	.32	.32	.32
Cognitive/Disorganization	20	4/7	**12**	2.4/7	**12**	2.4/7	**12**	2.4/7	**2.88***	**2.88***	**2.88***
Depression/Anxiety	8	2/7	7	1.8/7	7	1.8/7	7	1.8/7	.49	.49	.49
Excitability/Hostility	8	2/7	**5**	1.3/7	**4**	1/7	**4**	1/7	1.40	**1.86***	**1.86***

* RCI ≥ 1.64

GAF = Global Assessment of Functioning; PANSS = Positive and Negative Syndrome Scale

[a] PANSS RCIs are multiplied by -1. Therefore, a significant RCI indicates a decrease in the severity of the clinical symptoms.

Scores in bold for the PANSS indicate a clinical change after the treatment (a change of at least 25% compared to baseline). For the GAF, a score in bold indicates that the patient reached the remission criteria (59)

Table S4. ToM, cognition and metacognition raw scores, percentile ranks and RCIs for Case B at baseline, post-treatment and the two follow-ups

	Baseline raw score	Post-treatment raw score	3 months raw score	1 year raw score	Baseline Percentile	Post – treatment percentile	3 months percentile	1 year Percentile	Baseline – post-treatment RCI	Baseline – 3 months RCI	Baseline – 1 year RCI
A. Social cognition											
ToM - Combined Stories Task (/52)	23	30	32	31	<.10	.10	1	.20	**1.77***	**2.27***	**2.02***
B. Cognition											
Working memory – Digit span	12	11	12	11	5	5	9	5	-.41	.00	-.41
Working memory – Spatial span	13	13	10	15	16	16	2	**25**	.00	-1.60	1.07
Episodic memory – CVLT delayed recall	6	9	7	5	2	16	7	1	1.60	.53	-.53
Episodic memory – RCFT delayed recall	7	16.5	14	19.5	1	4	1	14	**1.97***	1.45	**2.59***
Selective attention – CPT omission	1	1	3	NA	70	70	46	NA	.00	-.33	NA
Selective attention – CPT commission	27	17	23	NA	5	**36**	13	NA	**1.90***	.76	NA
Sustained attention – Hit reaction time Block change	.02	.01	.01	NA	23	32	43	NA	.53	.53	NA
Sustained attention – Hit standard error Block change	.13	.06	.04	NA	5	**19**	**29**	NA	1.32	**1.69***	NA
Reasoning – Matrix[a]	9	7	10	11	37	16	50	63	-1.22	.61	1.22
Cognitive flexibility – WCST – Total Categories	2	.00	.00	1	1	1	1	1	-1.46	-1.46	-.73
Inhibition – Stroop 3	48	40	46	48	63	84	63	63	.67	.17	.00
Planning / Organization – TOL – Total Correct	2	5	5	3	19	60	60	33	**1.89***	**1.89***	.63
Metacognition regulation – BRIEF self GEC[b]	54	44	46	53	33	72	66	37	1.01	.81	.10
Metacognition regulation – BRIEF informant GEC[b]	65	64	60	64	6	8	16	8	.13	.66	.13
Metacognition knowledge – SSTICS	15	25	20	34	81	52	66	27	-2.55	-1.28	-4.85

ToM = Theory of mind score of the Combined Stories Task; CVLT = California Verbal Learning Test –II; RCFT = Rey Complex Figure Test; CPT = Continuous Performance Test – II; WCST = Wisconsin Card Sorting Test -128 card; TOL = Tower Of London ; BRIEF – self GEC = Executive Global Index – self-report; BRIEF – informant GEC = Executive Global Index – informant report; SSTICS = Subjective Scale to Investigate Cognition in Schizophrenia

[a] Scaled scores with a mean of 10 and a standard deviation of 3.

[b] T scores with a mean of 50 and a standard deviation of 10.

Percentiles in bold indicate a clinical change after the treatment for the cognitive and metacognitive measures (a percentile that surpasses the 16th percentile compared to baseline)

* RCI ≥ 1.64

Table S5. Clinical information for Case C at baseline, post-treatment and the two follow-ups

	Case C										
	Baseline raw score	Baseline mean	Post-treatment raw score	Post-treatment mean	3 months raw score	3 months mean	1 year raw score	1 year mean	Baseline – post-treatment RCI	Baseline – 3 months RCI	Baseline – 1 year RCI
GAF	45	-	45	-	45	-	NA	-	.00	.00	NA
PANSS[a]											
Total	52	1.9/7	49	-	49	-	NA	-	.42	.42	NA
Positive	8	1.3/7	8	1.3/7	8	1.3/7	NA	NA	.00	.00	NA
Negative	22	3.1/7	20	2.9/7	20	2.9/7	NA	NA	.65	.65	NA
Cognitive/Disorganization	11	2.2/7	10	2/7	10	2/7	NA	NA	.36	.36	NA
Depression/Anxiety	7	1.8/7	7	1.8/7	7	1.8/7	NA	NA	.00	.00	NA
Excitability/Hostility	4	1/7	4	1/7	4	1/7	NA	NA	.00	.00	NA

* RCI ≥ 1.64

GAF = Global Assessment of Functioning; PANSS = Positive and Negative Syndrome Scale

[a] PANSS RCIs are multiplied by -1. Therefore, a significant RCI indicates a decrease in the severity of the clinical symptoms.

Scores in bold for the PANSS indicate a clinical change after the treatment (a change of at least 25% compared to baseline). For the GAF, a score in bold indicates that the patient reached the remission criteria (59)

Table S6. ToM, cognition and metacognition raw scores, percentile ranks and RCIs for Case C at baseline, post-treatment and the two follow-ups

	Baseline raw score	Post-treatment raw score	3 months raw score	1 year raw score	Baseline percentile	Post-treatment percentile	3 months percentile	1 year percentile	Baseline – post-treatment RCI	Baseline – 3 months RCI	Baseline – 1 year RCI
A. Social cognition											
ToM - Combined Stories Task (/52)	37	44	46	NA	8	51	51	NA	**1.77***	**2.27***	NA
B. Cognition											
Working memory – Digit span	28	15	17	NA	25	25	37	NA	-5.27	-4.46	NA
Working memory – Spatial span	15	13	11	NA	25	16	5	NA	-1.07	-2.14	NA
Episodic memory – CVLT delayed recall	11	10	11	NA	32	7	50	NA	-.53	.00	NA
Episodic memory – RCFT delayed recall	8	3	3.5	NA	1	1	1	NA	-1.04	-.93	NA
Selective attention – CPT omission	17	5	5	NA	1	**24**	**24**	NA	**1.99***	**1.99***	NA
Selective attention – CPT commission	27	23	20	NA	5	13	**23**	NA	.76	1.33	NA
Sustained attention – Hit reaction time Block change	-.02	.00	.01	NA	77	51	38	NA	-1.05	-1.58	NA
Sustained attention – Hit standard error Block change	.02	.06	.07	NA	44	22	19	NA	-.75	-.94	NA
Reasoning – Matrix[a]	4	3	10	NA	2	1	**50**	NA	-.61	**3.65***	NA
Cognitive flexibility – WCST – Total Categories	4	6	6	NA	11	**16**	**16**	NA	1.46	1.46	NA
Inhibition – Stroop 3	50	47	53	NA	50	63	37	NA	.25	-.25	NA
Planning / Organization – TOL – Total Correct	4	3	1	NA	47	33	8	NA	-.63	-1.89	NA
Metacognition regulation – BRIEF self GEC[b]	50	61	68	NA	50	14	3	NA	-1.11	-1.82	NA
Metacognition regulation – BRIEF informant GEC[b]	51	68	66	NA	47	3	5	NA	-2.23	-1.96	NA
Metacognition knowledge – SSTICS	2	11	8	NA	97	86	90	NA	-2.30	-1.53	NA

ToM = Theory of mind score of the Combined Stories Task; CVLT = California Verbal Learning Test –II; RCFT = Rey Complex Figure Test; CPT = Continuous Performance Test – II; WCST = Wisconsin Card Sorting Test -128 card; TOL = Tower Of London ; BRIEF – self GEC = Executive Global Index – self-report; BRIEF – informant GEC = Executive Global Index – informant report; SSTICS = Subjective Scale to Investigate Cognition in Schizophrenia

[a] Scaled scores with a mean of 10 and a standard deviation of 3.

[b] T scores with a mean of 50 and a standard deviation of 10.

Percentiles in bold indicate a clinical change after the treatment for the cognitive and metacognitive measures (a percentile that surpasses the 16th percentile compared to baseline)

* RCI ≥ 1.64

Table S7. Clinical information for Case D at baseline, post-treatment and the two follow-ups

	Case D										
	Baseline raw score	Baseline mean	Post-treatment raw score	Post-treatment mean	3 months raw score	3 months mean	1 year raw score	1 year mean	Baseline – post-treatment RCI	Baseline – 3 months RCI	Baseline – 1 year RCI
GAF	48	-	48	-	NA	NA	NA	NA	.00	NA	NA
PANSS[a]											
Total	71	2.7/7	62	-	NA	NA	NA	NA	1.26	NA	NA
Positive	13	2.2/7	14	2.3/7	NA	NA	NA	NA	-.38	NA	NA
Negative	24	3.4/7	22	3.1/7	NA	NA	NA	NA	.65	NA	NA
Cognitive/Disorganization	16	3.2/7	**12**	2.4/7	NA	NA	NA	NA	1.44	NA	NA
Depression/Anxiety	14	3.5/7	**10**	2.5/7	NA	NA	NA	NA	**1.98***	NA	NA
Excitability/Hostility	4	1/7	4	1/7	NA	NA	NA	NA	.00	NA	NA

* RCI ≥ 1.64

GAF = Global Assessment of Functioning; PANSS = Positive and Negative Syndrome Scale

[a] PANSS RCIs are multiplied by -1. Therefore, a significant RCI indicates a decrease in the severity of the clinical symptoms.

Scores in bold for the PANSS indicate a clinical change after the treatment (a change of at least 25% compared to baseline). For the GAF, a score in bold indicates that the patient reached the remission criteria (59)

Table S8. ToM, cognition and metacognition raw scores, percentile ranks and RCIs for Case D at baseline, post-treatment and the two follow-ups

	Baseline raw score	Post-treatment raw score	3 months raw score	1 year raw Score	Baseline percentile	Post-treatment percentile	3 months percentile	1 year percentile	Baseline – post-treatment RCI	Baseline – 3 months RCI	Baseline – 1 year RCI
A. Social cognition *ToM* - Combined Stories Task (/52)	39	44	47	50	16	51	75	91	1.26	**2.02***	**2.78***
B. Cognition											
Working memory – Digit span	16	18	20	20	37	50	75	75	.81	1.62	1.62
Working memory – Spatial span	20	18	18	16	84	63	63	50	-1.07	-1.07	-2.14
Episodic memory – CVLT delayed recall	14	14	16	16	70	70	94	93	.00	1.06	1.06
Episodic memory – RCFT delayed recall	11	20	22	17.5	1	**24**	**38**	10	**1.86***	**2.28***	1.35
Selective attention – CPT omission	0	1	1	.00	79	70	70	79	-.17	-.17	.00
Selective attention – CPT commission	11	14	10	16	63	48	71	35	-.57	.19	-.95
Sustained attention – Hit reaction time Block change	.05	.02	-.01	.04	3	**25**	**62**	9	1.58	**3.16***	.53
Sustained attention – Hit standard error Block change	.03	.00	-.03	.04	38	53	62	33	.56	1.13	-.19
Reasoning – Matrix[a]	9	13	10	13	37	84	50	84	**2.43***	.61	**2.43***
Cognitive flexibility – WCST – Total Categories	6	6	6	6	16	16	16	16	.00	.00	.00
Inhibition – Stroop 3	66	64	54	56	16	16	**50**	**37**	.17	1.00	.84
Planning / Organization – TOL – Total Correct	4	1	.00	5	40	9	1	51	-1.89	-2.52	.63
Metacognition regulation – BRIEF self GEC[b]	60	59	59	NA	16	**18**	**18**	NA	.10	.10	NA
Metacognition regulation – BRIEF informant GEC[b]	56	52	52	NA	27	45	45	NA	.52	.52	NA
Metacognition knowledge – SSTICS	41	36	37	NA	14	**19**	**18**	NA	1.28	1.02	NA

ToM = Theory of mind score of the Combined Stories Task; CVLT = California Verbal Learning Test –II; RCFT = Rey Complex Figure Test; CPT = Continuous Performance Test – II; WCST = Wisconsin Card Sorting Test -128 card; TOL = Tower Of London ; BRIEF – self GEC = Executive Global Index – self-report; BRIEF – informant GEC = Executive Global Index – informant report; SSTICS = Subjective Scale to Investigate Cognition in Schizophrenia

[a] Scaled scores with a mean of 10 and a standard deviation of 3.

[b] T scores with a mean of 50 and a standard deviation of 10.

Percentiles in bold indicate a clinical change after the treatment for the cognitive and metacognitive measures (a percentile that surpasses the 16th percentile compared to baseline)

* RCI ≥ 1.64
